# Primary Mammary Organoid Model of Lactation and Involution

**DOI:** 10.3389/fcell.2020.00068

**Published:** 2020-03-19

**Authors:** Jakub Sumbal, Aurelie Chiche, Elsa Charifou, Zuzana Koledova, Han Li

**Affiliations:** ^1^Department of Developmental and Stem Cell Biology, Cellular Plasticity and Disease Modelling, CNRS UMR 3738, Institut Pasteur, Paris, France; ^2^Department of Histology and Embryology, Faculty of Medicine, Masaryk University, Brno, Czechia

**Keywords:** 3D culture, fibroblast growth factor 2, involution, lactation, mammary gland, milk production, organoid, prolactin

## Abstract

Mammary gland development occurs mainly after birth and is composed of three successive stages: puberty, pregnancy and lactation, and involution. These developmental stages are associated with major tissue remodeling, including extensive changes in mammary epithelium, as well as surrounding stroma. Three-dimensional (3D) mammary organoid culture has become an important tool in mammary gland biology and enabled invaluable discoveries on pubertal mammary branching morphogenesis and breast cancer. However, a suitable 3D organoid model recapitulating key aspects of lactation and involution has been missing. Here, we describe a robust and straightforward mouse mammary organoid system modeling lactation and involution-like process, which can be applied to study mechanisms of physiological mammary gland lactation and involution as well as pregnancy-associated breast cancer.

## Introduction

Lactation, the production of milk to feed progeny, is achieved by the mammary gland. This hallmark organ of mammals mainly develops postnatally and is highly dynamic (Macias and Hinck, 2012). With each pregnancy, mammary epithelium undergoes massive proliferation, tertiary branching of the mammary ductal system, and alveoli differentiation to prepare the epithelium for proper lactation (Brisken and Rajaram, 2006; Sternlicht, 2006). After parturition, mammary epithelium fully transforms into a milk-producing factory. Alveoli expand and take up space of regressing mammary stromal adipocytes, thereby multiplying epithelial volume many times (Macias and Hinck, 2012). After weaning, when milk production is no longer required, milk-producing epithelial cells are removed, and mammary gland is remodeled into a prepregnancy state. This process is called involution, which includes programmed cell death of the epithelium, ECM remodeling, and redifferentiation of adipocytes (Hughes and Watson, 2012; Macias and Hinck, 2012; Zwick et al., 2018; Jena et al., 2019). By the end of involution, mammary gland is ready for a new cycle of pregnancy-associated growth, lactation, and subsequent involution, which can be repeated throughout the reproductive lifespan. During these changes, mammary epithelium retains its bilayered architecture with lumen-facing luminal cells and basally situated myoepithelial cells, which is essential for proper function of the organ (Adriance et al., 2005; Haaksma et al., 2011; Macias and Hinck, 2012).

Endocrine signaling is a crucial regulator of mammary morphogenesis during pregnancy. Ovarian hormones estrogen and especially progesterone govern growth and morphogenesis of epithelium via induction of paracrine signaling between mammary stroma and epithelium, involving members of several growth factor families (Hennighausen and Robinson, 2005; Brisken and O’Malley, 2010). Pituitary hormone prolactin, on the other hand, acts directly on prolactin receptor on luminal cells and triggers alveoli maturation and lactogenic differentiation (Hennighausen and Robinson, 2005; Brisken and Rajaram, 2006). Involution is linked to cessation of hormonal stimuli and increase in inflammatory cytokines (Watson, 2006; Stein et al., 2007).

To study various aspects of mammary gland biology, three-dimensional (3D) cell culture models have been widely used for decades (Koledova,2017a). They combine the advantages of easy manipulation of 2D cellular systems with providing complex cell–cell and cell–ECM interactions, thereby mimicking physiological conditions of *in vivo* experiments more faithfully (Shamir and Ewald, 2014; Huch and Koo, 2015; Koledova,2017a; Artegiani and Clevers, 2018). Among the 3D culture models, primary mammary organoids have played a major role in understanding mechanisms of mammary branching morphogenesis (Ewald et al., 2008; Huebner et al., 2016; Neumann et al., 2018), including the role of ECM (Simian et al., 2001) and stromal cells (Sumbal and Koledova, 2019). Furthermore, spheroids produced from mammary cell lines were used to study tissue response to growth factors (Xian et al., 2005); organoids grown from sorted single primary mammary epithelial cells were used to study developmental potential of mammary epithelial cells (Linnemann et al., 2015; Jamieson et al., 2017), and differentiation of mammary-like organoids was achieved from induced pluripotent stem cells (Qu et al., 2017).

Despite these advances in 3D cell culture models of mammary gland, systems faithfully modeling pregnancy-associated morphogenesis and lactation have been spare. In some studies, β-casein or milk protein expression was used as a read-out of mammary epithelial functionality (Mroue et al., 2015; Jamieson et al., 2017). Several aspects of lactation and involution were captured in a coculture of mammary epithelial and preadipocyte cell lines (Campbell et al., 2014) or in hormone-treated breast cancer cell spheroids (Ackland et al., 2003; Freestone et al., 2014). However, a system modeling lactation and involution in primary mammary organoids with proper architecture of bilayered epithelium with myoepithelial cell layer has not been characterized.

Here, we report on a mammary 3D culture system for studying induction and maintenance of lactation using easily accessible and physiologically relevant murine primary mammary organoids cultured in Matrigel. Upon prolactin stimulation, the organoids produce milk for at least 14 days and maintain a histologically normal architecture with a functional contractile myoepithelial layer. Moreover, upon prolactin signal withdrawal, our system recapitulates several aspects of involution. Altogether, we describe a robust, consistent, and easy-to-do system for modeling crucial aspects of pregnancy-associated mammary gland morphogenesis and lactation.

## Materials and Methods

### Isolation of Primary Mammary Epithelial Organoids

Primary mammary organoids were prepared from 7- to 10-week-old female mice (ICR or C57/BL6) as previously described (Koledova,2017b; Supplementary Figure 1A). ICR strain was used for the branching morphogenesis and time-lapse imaging, cell viability and replating assays, and confocal imaging. C57/BL6 strain was used for the rest of the experiments. The animals were obtained from the Central Animal Facility of the Institut Pasteur and the Laboratory Animal Breeding and Experimental Facility of the Faculty of Medicine, Masaryk University. Experiments involving animals were approved in accordance with French legislation in compliance with European Communities Council Directives (A 75-15-01-3), the regulations of Institut Pasteur Animal Care Committees (CETEA), the Ministry of Agriculture of the Czech Republic, and the Expert Committee for Laboratory Animal Welfare at the Faculty of Medicine, Masaryk University. The study was performed by certified individuals (AC, JS, EC, and ZK) and carried out in accordance with the principles of the Basel Declaration.

Briefly, the mice were euthanized by cervical dislocation, the thoracic and inguinal mammary glands were collected, visible lymph nodes were excised, and the pooled mammary glands were finely chopped to approximately 1-mm^3^ pieces and digested in a solution of collagenase and trypsin [2 mg/mL collagenase (Roche, Switzerland or Sigma, United States), 2 mg/mL trypsin (^∗^Dutscher Dominique, France or Sigma, United States), 5 μg/mL insulin (Sigma, United States), 50 μg/mL gentamicin (Sigma, United States), 5% fetal bovine serum (Hyclone/GE Healthcare, United States) Dulbecco’s in modified Eagle medium (DMEM)/F12 (Thermo Fisher Scientific, United States)] for 30 min at 37°C with shaking at 100 rpm. Next, the tissue suspension was treated with 20 U/mL DNase I (Sigma, United States) and 0.5 mg/mL dispase II (Roche, Switzerland) and exposed to five rounds of differential centrifugation at 450 × *g* for 10 s, which resulted in separation of epithelial (organoid) and stromal fractions (Supplementary Figure 1A). The organoids were resuspended in basal organoid medium [BOM; 1× insulin–transferrin–selenium supplement, 100 U/mL of penicillin, and 100 μg/mL of streptomycin, in DMEM/F12 (all from Thermo Fisher Scientific, United States)] and kept on ice up to 2 h before seeding for 3D culture.

### 3D Culture of Mammary Organoids

Freshly isolated primary mammary organoids were mixed with growth factor reduced Matrigel (Corning, United States) and plated in domes in 24-well culture plate (one dome per well, 70 μL of Matrigel per dome). 200, 400, or 1000 organoids per dome were seeded for histology, gene expression, and Western blot analysis, respectively. After setting the Matrigel for 45–60 min at 37°C, the 3D organoid cultures were overlaid with cell culture medium according to the experiment and incubated at 37°C in a humidified atmosphere with 5% CO_2_ (Supplementary Figure 1B). The media used were as follows: growth factor medium [BOM supplemented with different growth factors: 2.5 nM FGF2 (Peprotech, United States or Thermo Fisher Scientific, United States), 2.5 nM FGF7, 2.5 nM FGF10, 50 ng/mL EGF (all from Peprotech, United States), 5 nM TGFα (Sigma, United States), or a combination of 10 ng/mL WNT3A and 50 ng/mL R-spondin 1 (W3/R1, both from Peprotech, United States)] and lactation medium {LM; 1 μg/mL prolactin [mouse recombinant prolactin for quantitative polymerase chain reaction (qPCR), Western blot, immunohistochemistry and contraction experiments (Sigma, United States or Peprotech, United States), and sheep pituitary prolactin for confocal and time-lapse imaging, including contraction experiments (Sigma, United States)], and 1 μg/mL hydrocortisone (Sigma, United States) in BOM}. Media containing growth factors were changed every 3 days; LM was changed every 2 days. To induce contraction of lactation organoids grown with mouse recombinant prolactin, 40 μg/mL recombinant oxytocin (Sigma, United States) was used. For time-lapse imaging experiments, organoid cultures were incubated in a humidified atmosphere of 5% CO_2_ at 37°C on Olympus IX81 microscope equipped with Hamamatsu camera and CellR system for time-lapse imaging. For morphological analysis of organoid development, the organoids were photographed from days 8 to 17 of culture; one image per organoid was taken every hour. The images were exported and analyzed using ImageJ (NIH, United States). For analysis of organoid contraction, the organoids were photographed from days 6 to 20 of culture. On each imaging day, the photographs were taken every second for 120 s. The images were exported to video at 10 frames per second using xCellence software (Olympus, Japan).

### Replating of Organoids

To replate organoids, 3D cultures were rinsed with phosphate-buffered saline (PBS) and disintegrated by pipetting up and down in ice-cold PBS with a 1000 μL pipette. Successful disintegration of Matrigel was checked under a microscope. Organoid suspensions were centrifuged at 450 × *g* for 3 min. Organoid pellets were resuspended in fresh Matrigel and plated as described above. Organoids were maintained in BOM or in BOM supplemented with 2.5 nM FGF2; the medium was changed every 3 days. Organoid area was measured in ImageJ.

### Cell Viability Assay

To asses cell viability in organoids treated with LM or LM-BOM, on the 20th day of culture, the media were changed with fresh BOM, and then resazurin (Merck, Germany) was added to the medium to the final concentration of 10 μg/mL. The plates were incubated for 6 h. Resorufin fluorescence (excitation at 560 nm, emission at 590 nm) was measured using Synergy H4 Hybrid multimode microplate reader (BioTek, United States) in technical triplicates. As a positive control of dying cells, organoids in LM-BOM conditions were treated from day 16 with 40 μM taxol (Sigma, United States) or killed on day 20 by treatment with 70% ethanol for 5 min.

### Histology and Immunostaining Analysis

For histological analysis, organoids were fixed in 4% paraformaldehyde (Electron Microscopy Sciences, United States) for 30 min and embedded in 3% low gelling temperature agarose (Supplementary Figure 1C). After solidification, samples were dehydrated and paraffin embedded and cut in 5-μm sections, which were dewaxed for hematoxylin and eosin staining or immunostaining. For localization of prolactin receptor expressing cells, 10-μm cryosections of mammary glands from *Prlr-IRES-Cre;ROSA26-CAGS-GFP* mice (Aoki et al., 2019) were labeled with antibodies and counterstained with 0.5 μg/mL DAPI, mounted with Vectashield (Vector Labs, United States), and images were taken on LSM800 microscope (Zeiss, Germany). The following primary antibodies were used: goat anti-GFP (Origene, United States, R1091P, 1:200), rabbit polyclonal anti-keratin 5 (BioLegend, United States, 905501, 1:200), mouse monoclonal anti-keratin 8 (BioLegend, United States, 904801, 1:200), mouse monoclonal anti-β-casein (Santa Cruz, United States, sc-166530, 1:250), and rabbit anti-mouse milk proteins (^∗^Accurate Chemical, United States, YNRMTM, 1:500). Corresponding secondary antibodies were used: donkey anti-rabbit Dylight 488 (Immuno Reagents, United States, DkxRb-003-D594NHSX, 1:200) and donkey anti-mouse Dylight 594 (Immuno Reagents, United States, DkxMu-003-D488NHSX, 1:200), together with 1 μg/mL of Hoechst-33342 (Thermo Fisher Scientific, United States) for immunofluorescence labeling, or anti-mouse/anti-rabbit horseradish peroxidase (HRP)-associated secondary antibodies (Dako, United States).

### Whole Mount Staining of Mammary Organoids

Organoids were fixed with 10% formalin for 30 min, washed with PBS and 70% ethanol, and incubated with oil red O solution [0.3% (wt/vol) oil red O (Sigma, United States) in 70% (vol/vol) ethanol (Koopman et al., 2001; Kim et al., 2015)] for 30 min in the dark. Next, organoids were washed with 70% ethanol and PBS and incubated with 0.5 μg/mL DAPI and 2 units/sample phalloidin-AlexaFluor488 (Thermo Fisher Scientific, United States) in PBS for 1 h at room temperature (RT) in the dark. Subsequently, organoids were washed and transferred to coverslip-bottom 35-mm dishes (ibidi) covered with 1% low gelling temperature agarose (Sigma, United States) and overlaid with PBS. Images were acquired using LSM800 confocal microscope (Zeiss, Germany, Supplementary Figure 1D) and analyzed using ZEN blue software (Zeiss, Germany).

### RNA Isolation and Real-Time qPCR

Total RNA was extracted from organoid samples using RNeasy Micro Kit (Qiagen, Germany) following the manufacturer’s instructions. Reverse transcription was performed using high-capacity cDNA reverse transcription kit (Thermo Fisher Scientific, United States). Quantitative real-time PCR was performed using 5 ng cDNA, 5 pmol of the forward and reverse gene-specific primers each in Light Cycler SYBR Green I Master mix (Roche, Switzerland) on LightCycler 480 II (Roche, Switzerland). All reactions were performed at least in duplicates and in a total of at least two independent assays. Relative gene expression was calculated using the ΔΔCt method, and the values were normalized to housekeeping gene *Gapdh*. The primers of following sequences (5′–3′) were used: *Csn2*-forward (F): CCTCTGAGACTGATAGTATTT, *Csn2*-reverse (R): TGGATGCTGGAGTGAACTTTA; *Wap*-F: TT GAGGGCACAGAGTGTATC, *Wap*-R: TTTGCGGGTCCTACC ACAG; *Mmp3*-F: CCTGATGTTGGTGGCTTCA, *Mmp3*-R: TC CTGTAGGTGATGTGGGATTTC; *Mmp13*-F: ACTTCTACCCA TTTGATGGACCTT, *Mmp13*-R: AAGCTCATGGGCAGCAA CA; *Gapdh*-F: TTCACCACCATGGAGAAGGC, *Gapdh*-R: CC CTTTTGGCTCCACCCT. All primers were purchased from Sigma, United States.

### Western Blot

Three-dimensional cultures were dissociated by repetitive pipetting in ice-cold PBS supplied with phosphatase inhibitor cocktail II (Merck, Germany; 2 mM imidazole, 1 mM sodium fluoride, 1.15 mM sodium molybdate, 1 mM sodium orthovanadate, 4 mM sodium tartrate dihydrate), followed by centrifugation at 450 × *g* for 3 min at 4°C. Supernatant was discarded, and pellets were lysed in ready-to-use RIPA buffer [Merck, Germany; 150 mM NaCl, 1.0% IGEPAL^®^ CA-630, 0.5% sodium deoxycholate, 0.1% sodium dodecyl sulfate (SDS), 50 mM Tris, pH 8.0] supplied with protease inhibitor cocktail I (Merck, Germany; 500 μM AEBSF hydrochloride, 150 nM aprotinin, 1 μM protease inhibitor E-64, 0.5 mM EDTA, 1 μM leupeptin hemisulfate) and phosphatase inhibitor cocktail II. After vortexing and sonication, protein lysates were cleared by centrifugation, and protein concentration was measured using Coomassie reagent (Merck, Germany). Denatured, reduced samples were resolved on 12.5% SDS–polyacrylamide electrophoresis (Bio-Rad, United States) and blotted onto nitrocellulose membranes by Trans-blot Turbo transfer system (Bio-Rad, United States). After blotting, the membranes were blocked with 2% bovine serum albumin in PBS with 0.1% Tween-20 (Merck, Germany; blocking buffer) and incubated with primary antibodies diluted in blocking buffer overnight at 4°C. After washing in PBS with 0.05% Tween-20, the membranes were incubated with HRP-conjugated secondary antibodies for 1 h at RT. Signal was developed using an ECL substrate (Thermo Fisher Scientific, United States) and imaged with ChemiDoc MP imaging system (Bio-Rad, United States), and band density was analyzed in ImageJ. The following antibodies were used for immunoblotting: mouse monoclonal anti-β-casein (Santa Cruz, United States, sc-166530, 1:1000), mouse monoclonal anti-α-tubulin (Santa Cruz, United States, sc-5286, 1:1000), and anti-mouse secondary antibody (Merck, NA931, 1:1000).

### Statistical Analysis

Statistical analysis was performed using the Prism software (GraphPad, United States); statistical test used is specified in figure legends. ^∗^*p* < 0.05, ^∗∗^*p* < 0.01, ^∗∗∗^*p* < 0.001, ^****^p < 0.0001. The number of independent biological replicates is indicated as *n*.

## Results

### FGF2 Pretreatment Enhances Lactogenic Differentiation of Mammary Epithelium

During mammary gland morphogenesis, lactation is preceded by excessive branching of epithelial ducts. We hypothesized that epithelial expansion by branching morphogenesis might be required for lactogenic differentiation *in vitro*. Therefore, we first tested the impact of several growth factors on mammary epithelial morphogenesis. The primary mammary epithelial organoids were treated with FGF2, FGF7, FGF10, EGF, TGFα, or a combination of WNT3A and R-spondin 1 (W3/R1) for 7 days. Interestingly, only FGF2, a potent mammary epithelium branching-inducing factor (Ewald et al., 2008), induced extensively branched morphology (Supplementary Figures 2A–D).

**FIGURE 1:**
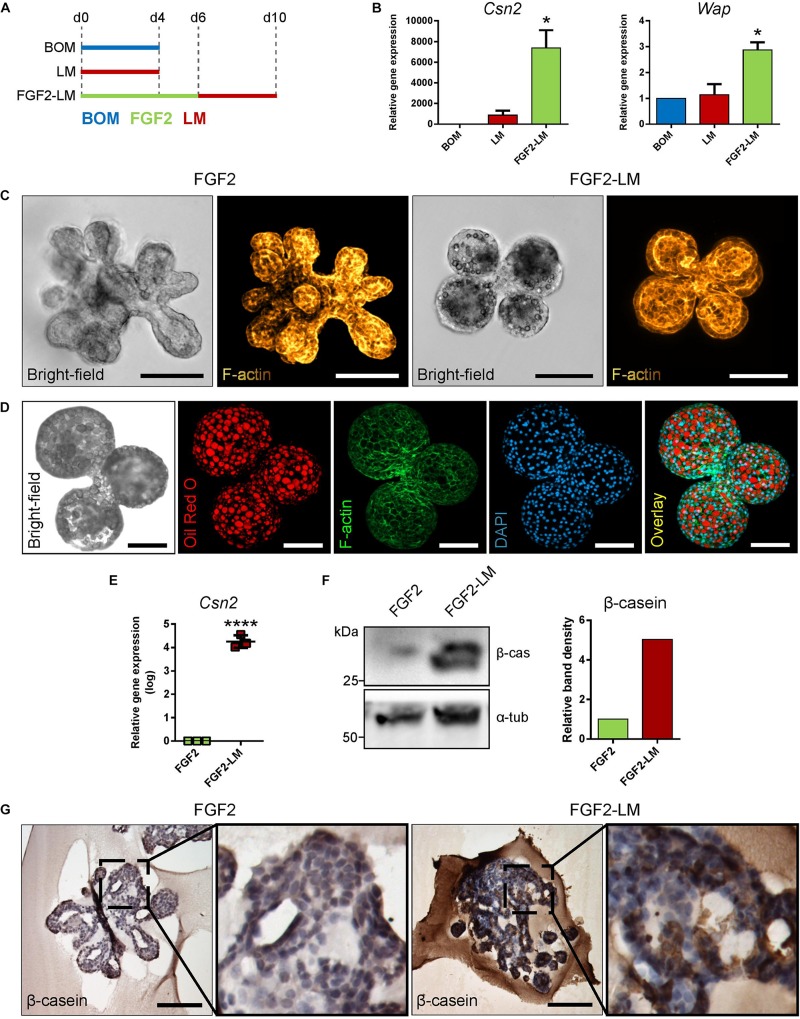
Lactation induction in primary mammary organoids. **(A,B)** FGF2 pretreatment increases lactation capacity of primary mammary organoids. **(A)** Scheme depicting the experimental design. BOM, basal organoid medium; LM, lactation medium; FGF2, FGF2 medium. **(B)** Expression of milk genes *Csn2* and *Wap* in organoids treated with BOM, LM, or FGF2 followed by LM. The values are relative to BOM. The plot shows mean + SD; *n* = 2. One-way ANOVA, *p < 0.05. **(C)** Bright-field images and maximum intensity projection images from confocal imaging of whole-mount organoids after treatment with FGF2 only or with FGF2 followed by LM. Yellow-to-brown staining shows F-actin. Scale bars represent 100 μm. **(D)** Bright-field image and maximum intensity projection images from confocal imaging of whole-mount organoid treated with FGF2 followed by LM. Red, oil red O (lipids); green, F-actin; blue, DAPI (nuclei). Scale bars represent 100 μm. **(E,F)** Quantification of β-casein expression in organoids treated with FGF2, or FGF2 followed by LM. **(E)** RT-qPCR analysis of β-casein gene *Csn2*. The values are relative to FGF2. The plot shows mean ± SD; *n* = 3. Unpaired Student’s *t*-test, two tailed, ****p < 0.0001. **(F)** Western blot analysis of β-casein expression on protein level. The plot shows quantification of band density. The values are relative to FGF2. **(G)** Immunohistochemical staining of β-casein in organoids treated with FGF2 or FGF2 and then LM at days 6 and 10, respectively. Marked area is shown in higher magnification. Scale bars represent 100 μm.

Next, we tested if FGF2-induced epithelial expansion facilitated lactogenic differentiation. To this end, the primary mammary epithelial organoids were either treated only with LM (containing prolactin and hydrocortisone) for 4 days, or they were treated with FGF2 for 6 days and followed by 4 days of LM (Figure 1A). To detect lactogenic differentiation, we measured the expression of *Csn2* and *Wap* by RT-qPCR. Our results revealed that treatment of freshly isolated organoids with LM induced only expression of *Csn2* (Figure 1B). However, when organoids were pretreated with FGF2, the expressions of both *Csn2* and *Wap* were significantly increased (Figure 1B). These data suggest that mammary epithelial expansion, induced by branching morphogenesis, could enhance the lactogenic ability of mammary epithelium.

### Lactation Medium Induces Production of Milk Proteins and Secretion of Lipid Droplets

Next, we compared the morphology of organoids treated with either FGF2 only or FGF2 and LM (FGF2-LM) to further characterize the phenotype of lactation organoids. On bright-field micrographs, we noticed that FGF2-LM organoids appeared to have a darker lumen, possibly due to the milk accumulation (Figure 1C). Interestingly, we also observed bubble-like structures at the apical site of epithelium in the same organoids, which potentially represented lipid droplets (Figure 1C). To further characterize these droplets, we stained the organoids for F-actin (with phalloidin), a cytoskeletal protein, or with oil red O. Confocal microscopy revealed that the droplets were negative for F-actin and strongly positive for oil red O, confirming the droplets were lipid (Figures 1C,D).

Next, we assessed the expression of milk proteins in the organoids. First, we detected a significant increase in *Csn2* by four orders in FGF2-LM-treated organoids compared to FGF2 alone by RT-qPCR (Figure 1E). Consistently, in FGF2-LM-treated organoids, we detected up-regulation of β-casein on the protein level by Western blot (Figure 1F) and a strong cytoplasmic signal by immunohistochemistry (Figure 1G), which was further confirmed by antibody against milk proteins (Supplementary Figures 3A–C). Taken together, these data demonstrate that mammary primary organoids are capable of milk production after prolactin treatment, which could be greatly enhanced by branching morphogenesis.

### Morphology Maintenance in Long-Term Lactating Organoids

**FIGURE 2:**
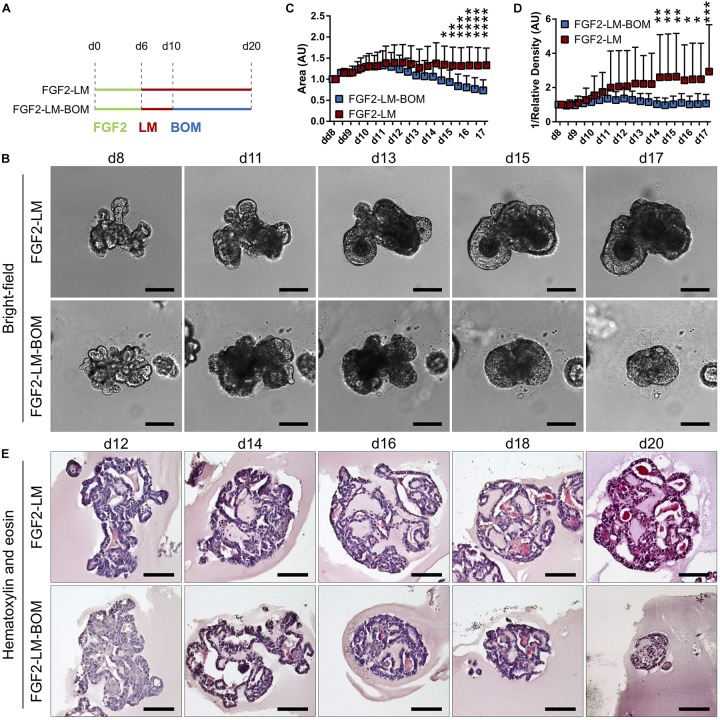
Morphology of organoids undergoing long-term lactation. **(A)** Scheme depicting experimental design. FGF2, FGF2 medium; LM, lactation medium; BOM, basal organoid medium. **(B)** Bright-field images from time-lapse imaging of organoid morphogenesis under continuous LM treatment (FGF2-LM) or under LM withdrawal and replacement with BOM (FGF2-LM-BOM). Scale bars represent 100 μm. **(C,D)** Morphometric analysis of organoid area **(C)** and density **(D)** from the time-lapse experiment. The plots show mean + SD; *n* = 2, *N* = 20 organoids per condition. Two-way ANOVA, *p < 0.05, **p < 0.01, ***p < 0.001, ****p < 0.0001. **(E)** Hematoxylin and eosin staining of organoids at different time points of long-term lactation. Scale bars represent 100 μm.

After successful induction of lactation in the primary mammary organoids with the FGF2-LM protocol, we went on to investigate the lactation-associated phenotype in long-term organoid culture. After 6 days of FGF2 treatment, the organoids were either cultured continuously with LM (FGF2-LM) or switched to BOM after 4 days of LM treatment (FGF2-LM-BOM) (Figure 2A). The morphogenesis of the organoids was recorded using time-lapse microscopy for 20 days. Interestingly, FGF2-LM-BOM cultured organoids regressed both in size and the complexity of the shape, whereas the organoids in FGF2-LM maintained the size and only partially lost the branched phenotype (Figures 2B,C and Supplementary Figures 4A,B). In contrast, continuous treatment with FGF2 for 20 days maintained the organoid branched morphology (Supplementary Figures 4A,B). In addition, unlike the organoids in FGF2-LM-BOM, the organoids in FGF2-LM retained the darker appearance, possibly due to the milk accumulation (Figures 2B,D and Supplementary Figure 4A). Morphologically, FGF2-LM-treated organoids exhibited complex architecture with multiple lumens filled with dense eosinophilic material, which was maintained throughout the experiment (Figure 2E, upper panel). However, upon LM withdrawal, the complex architecture was lost rapidly, and organoids involuted into small spheroids with much simpler structures (Figure 2E, lower panel).

### Milk Production in Long-Term Lactating Organoids

**FIGURE 3:**
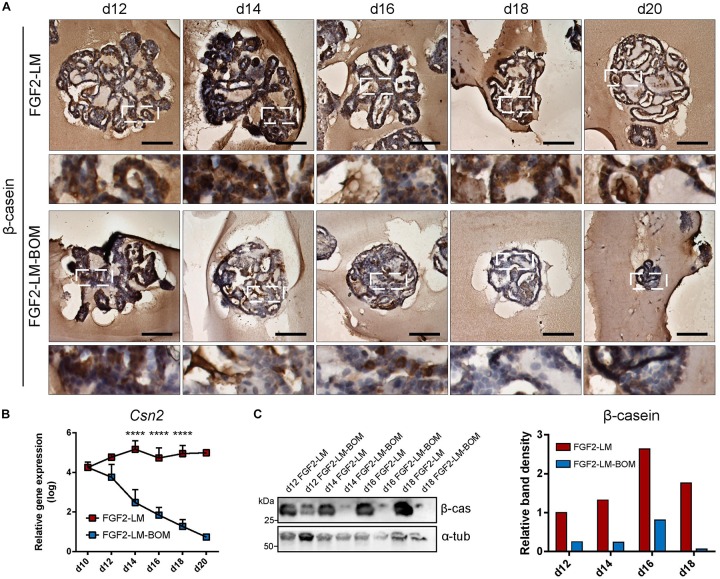
Milk production during long-term lactation. **(A)** Immunohistochemical staining of β-casein in organoids during long-term LM treatment or after LM withdrawal (LM-BOM), according to experimental scheme in Figure 2A. Marked area is shown in higher magnification. Scale bars represent 100 μm. **(B)** *Csn2* expression during long-term lactation with continuous lactation medium (FGF2-LM) or with hormonal withdrawal (FGF2-LM-BOM). The plot shows mean + SD; *n* = 3 for d12 to d18, *n* = 1 for d20. Two-way ANOVA, ****p < 0.0001. **(C)** Western blot analysis and band density quantification of β-casein expression in organoids during long-term lactation.

Of note, we detected strong β-casein signal in the intraluminal of long-term lactating organoids by immunohistochemistry. Closer observation revealed that cytoplasmic β-casein signal was sustained in long-term LM culture (Figure 3A, upper panel), but lost after LM withdrawal (Figure 3A, lower panel). In addition, RT-qPCR revealed that FGF2-LM-treated organoids maintained a high level of *Csn2* expression, which was dramatically reduced by four to five orders of magnitude in FGF2-LM-BOM-treated organoids (Figure 3B). The same change was confirmed in the protein level by Western blot (Figure 3C). Therefore, the production of β-casein depended on the prolactin signaling.

Altogether, these data suggest that these organoids have a proper epithelial architecture and the capacity to maintain milk production over prolonged culture time in response to the prolactin signaling.

### Lactating Organoids Retain Functional Myoepithelial Layer With Contractility

**FIGURE 4:**
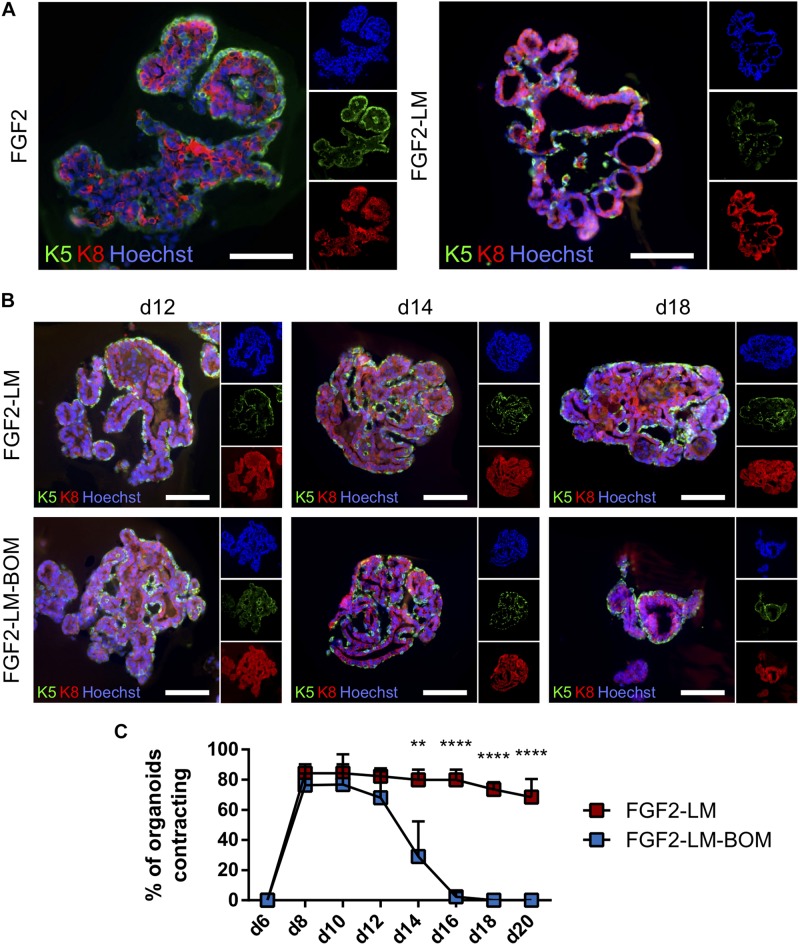
Lactating organoids retain functional myoepithelial layer. **(A)** Immunofluorescent staining shows distribution of myoepithelial (keratin 5 positive, green) and luminal cells (keratin 8 positive, red) in organoids treated with FGF2 or FGF2 followed by LM. Hoechst, blue (nuclei). Scale bars represent 100 μm. **(B)** Immunofluorescent staining shows distribution of myoepithelial (keratin 5 positive, green) and luminal cells (keratin 8 positive, red) in organoids during long-term lactation. Hoechst, blue (nuclei). Scale bars represent 100 μm. **(C)** Quantification of contracting organoids from movies recorded at indicated time-points. The plot shows mean + SD; *n* = 2, *N* = 50 organoids per experiment. Two-way ANOVA, **p < 0.01, ****p < 0.0001.

Next, we co-stained the lactating organoids for keratin 5 and keratin 8, markers of myoepithelial and luminal cells, respectively, to confirm that the organoids contain proper bilayer epithelial architecture. We found that FGF2-LM-treated organoids contained a continuous layer of myoepithelial cells, similar to FGF2-treated organoids (Figure 4A). Moreover, the myoepithelial cell layer was retained during the long-term culture in LM treatment, as well as after LM withdrawal (Figure 4B), suggesting the luminal–myoepithelial cell homeostasis was stable during long-term culture.

Importantly, FGF2 treatment induced stratification of the luminal layer, which is in agreement with published work (Figure 4A; Ewald et al., 2008). Upon LM treatment, the organoids showed resolution of the stratified epithelium to a predominantly bilayer structure, with luminal cells (keratin 8 positive) lining the luminal space (Figures 4A,B), which is important for producing milk. Remarkably, we observed the LM-treated organoids could contract periodically (Supplementary Movie 2). In comparison, organoids never treated with LM showed relatively static structures (Supplementary Movie 1). Of note, the contracting phenotype maintained during the long-term LM treatment and quickly ceased after LM withdrawal (Figure 4C). This result is somewhat puzzling because prolactin receptor is present only in the luminal cells (Supplementary Figure 5A). Of note, the prolactin used in our contraction experiments was isolated from sheep pituitary, which contains oxytocin (Vorherr et al., 1978). To test whether the contraction of myoepithelial cells is a direct effect of prolactin signaling, we compared contraction induction upon LM containing either sheep pituitary prolactin or mouse recombinant prolactin. Interestingly, only sheep pituitary prolactin induced organoid contraction; mouse recombinant prolactin did not induce contraction (Supplementary Figure 5B and Supplementary Movie 3). However, when the organoids cultured with mouse recombinant prolactin were treated with recombinant oxytocin, they did contract (Supplementary Movie 4), demonstrating that oxytocin is required for myoepithelial cell contraction. Taken together, these results demonstrate that myoepithelial layer is present in the lactating organoids. And more importantly, these myoepithelial cells can contract in response to LM treatment, suggesting they are functionally similar to the *in vivo* counterpart.

### LM Withdrawal Triggers Involution-Like Phenotype in Lactating Organoids

**FIGURE 5:**
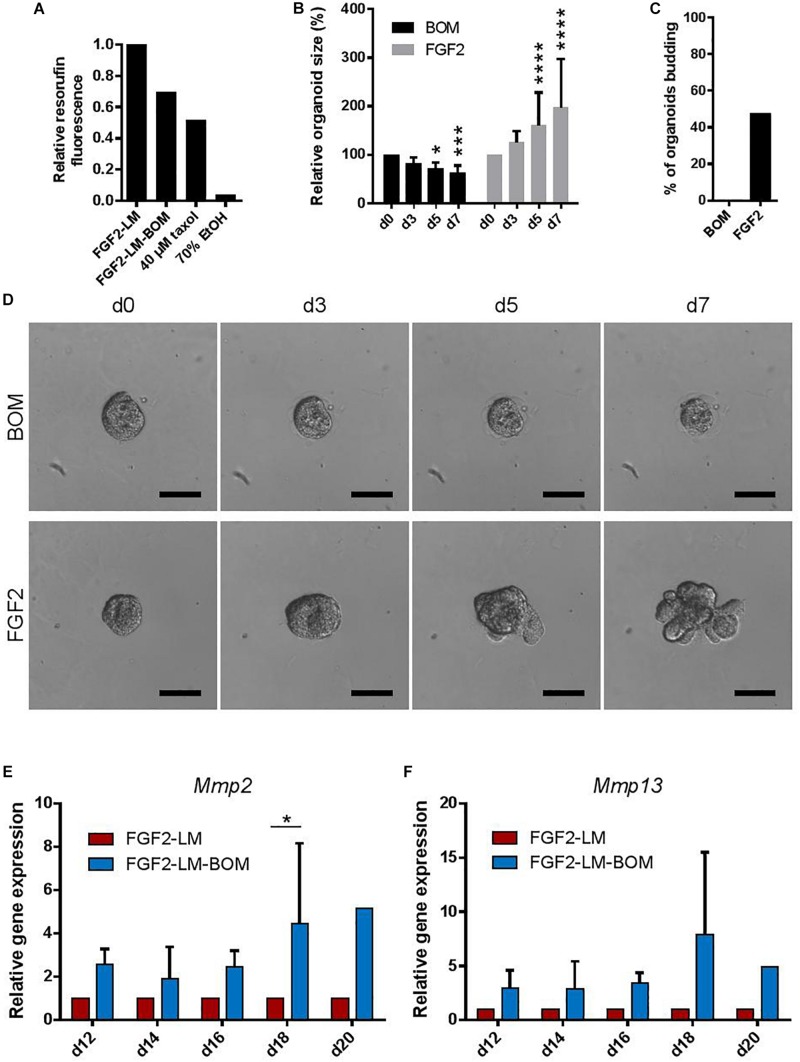
Withdrawing hormones induces an involution-like phenotype in lactating organoids. **(A)** The viability of the lactating and involuting organoids using resazurin assay. The plot shows relative resorufin fluorescence of organoids with continuous LM treatment (FGF2-LM), LM withdrawal and replacement with BOM (FGF2-LM-BOM), and FGF2-LM-BOM organoids treated with 40 μM taxol for 4 days (40 μM taxol) or 70% ethanol for 5 min (70% EtOH) to induce cell death. Values are relative to FGF2-LM. **(B–D)** Analysis of FGF2-LM-BOM organoids after replating to BOM or FGF2 medium. **(B)** Quantification of the size of the FGF2-LM-BOM organoids that were replated and cultured with BOM or FGF2 for the number of days as indicated. The plot shows mean + SD; *n* = 1, *N* = 25 organoids per condition. Two-way ANOVA, asterisks indicate change in comparison to d0; *p < 0.5, ***p < 0.001, ****p < 0.0001. **(C)** Quantification of the number of budding FGF2-LM-BOM organoids after replating and culture with BOM or FGF2 for 7 days. **(D)** Bright-field images showing morphogenesis of FGF2-LM-BOM organoids after replating and culture with BOM or FGF2 for 7 days. Scale bars represent 100 μm. **(E,F)** RT-qPCR analysis of *Mmp2* and *Mmp13* expression in organoids during long-term lactation with continuous lactation medium (LM) treatment or with hormonal/LM withdrawal (LM-BOM). The values are relative to FGF2-LM at each time point. The plots show mean + SD; *n* = 3 for d12–d18, *n* = 1 for d20. Two-way ANOVA, ^∗^*p* < 0.05.

Involution is characterized by the regression of the lactating epithelium through programmed cell death and remodeling of the mammary gland, which is induced upon weaning of the pups (Jena et al., 2019). Interestingly, withdrawal of LM from lactating organoids also induced a size regression and loss of the branched morphology with luminal architecture (Figures 2B–E). Using cell viability assay that is based on conversion of non-fluorescent resazurin to fluorescent resorufin by viable cells, we found that lactating organoids upon LM withdrawal (FGF2-LM-BOM) showed reduced viability in comparison to lactating organoids in LM (FGF2-LM) (Figure 5A), most likely due to increased cell death in response to LM withdrawal, which is a characteristic of involution. Yet the viability of organoids upon LM withdrawal was higher than that of organoids undergoing taxol- or ethanol-induced cell death (Figure 5A). Furthermore, replating of the involution-like organoids (FGF2-LM-BOM) to fresh Matrigel and FGF2 treatment reversed the size regression (Figure 5B) and, more importantly, induced branching morphogenesis (Figures 5C,D). This demonstrates that involuting organoids are viable and that the morphological changes induced upon LM withdrawal are reversible.

Furthermore, cessation of milk production and ECM remodeling are two hallmarks of involution. Consistently, we detected a reduced β-casein signal (Figures 3A,C) and *Csn2* expression (Figure 3B) in the organoids upon LM withdrawal. Interestingly, we also found that the expression of *Mmp2* and *Mmp13*, two important Mmps for the ECM remodeling process during involution, was up-regulated in organoids after LM withdrawal (Figures 5E,F). Together, these results demonstrate that upon withdrawal of hormonal stimulation lactating organoids stop milk production and enter an involution-like process, thereby mimicking the *in vivo* situation upon weaning.

## Discussion

In this work, we described the use of primary mammary epithelial organoids to model pregnancy-associated morphogenesis and lactation. In our 3D culture system, primary mammary organoids exposed to LM with prolactin recapitulated several aspects of lactation process. Upon LM withdrawal, organoids regressed in a manner similar to the involution process *in vivo*.

Our data showed that FGF2 primes mammary epithelium for lactation. This is consistent with *in vivo* studies that noted morphological abnormalities in pregnancy-associated tertiary branching of mammary epithelium with attenuated FGF receptor signaling (Lu et al., 2008; Parsa et al., 2008). However, it remains to be elucidated what of the FGF2-mediated processes, including epithelial expansion, branching, and maturation, are essential contributors to milk production efficiency.

While several previous studies reported lactation induction in mammary epithelial organoids in response to prolactin *in vitro*, they did so only at a single time point (Mroue et al., 2015; Jamieson et al., 2017). Long-term lactation in organoid cultures has not been reported before. In this study, we documented milk production maintenance and stable morphology of lactating organoids over 14 days’ culture period. Physiological lactation in mouse lasts for circa 3 weeks (König and Markl, 1987), and milk composition and production rate vary during the lactation period to accommodate the needs of the offspring (Knight et al., 1986). We propose that our model would be suitable to study factors that influence dynamic changes in milk composition and quantity in the long term. Among others, insulin is used in our model to support cell survival and growth and has been implicated in milk production (Nommsen-Rivers, 2016) both in rodent and human. Our model could help to further elucidate how insulin signaling impacts on milk production. Moreover, while previous studies used sample-destructive methods to detect lactation, such as organoid fixation and immunodetection of milk proteins (Mroue et al., 2015; Jamieson et al., 2017), we propose approaches for observing changes in milk production in the same organoid over time. They include morphological changes accompanying lactation in organoids, namely, appearance of lipid droplets in luminal space, increase in organoid darkness (integrated density), and the intriguing contraction of myoepithelial cells, which are easily observable by light microscopy and traceable by time-lapse imaging.

Myoepithelial cells form a layer of mammary epithelium that is situated basally to the luminal cells (Macias and Hinck, 2012). Besides the recently elucidated role in keeping epithelial homeostasis and integrity (Adriance et al., 2005; Goodwin and Nelson, 2018; Sirka et al., 2018), the key function of myoepithelial cells is to enable milk ejection by contraction when pups are suckling (Haaksma et al., 2011). In response to tactile stimuli, oxytocin is released from pituitary, and it binds to oxytocin receptor on myoepithelial cell to induce contraction (Nishimori et al., 1996; Froemke and Carcea, 2017). Therefore, oxytocin was used to induce myoepithelial contraction in single cells (Raymond et al., 2011), as well as in an organoid system (Mroue et al., 2015). However, organoid contraction was shown only as a decrease in organoid area over 20 min (Mroue et al., 2015). In contrary, we observed that contraction of a lactating organoid is a very fast process, and the dynamic changes in organoid shape and size are visible to human eye. From videos of contracting organoids, recorded at the rate of one frame per second, we calculated that the frequency is about one contraction per 10 s, which is very similar to the recently reported alveoli warping frequency of lactating mammary tissue upon oxytocin stimulation (Stewart et al., 2019). Therefore, our model provides a suitable *in vitro* system for studying the regulation of the contractile function of myoepithelial cells.

Upon LM withdrawal, lactating organoids underwent involution-like process: regression in size and complexity, which is reversible by FGF2 treatment upon reseeding; and up-regulation of the expression of MMPs, the proteases typically found in mammary gland during involution (Lund et al., 1996; Green and Lund, 2005). Involution-like morphological changes upon prolactin withdrawal were documented also in the 3D coculture model of lactation using mammary epithelial and preadipocyte cell lines. However, epithelial cells cultured without preadipocytes were not reported (Campbell et al., 2014). Thus, for the first time in organoid culture, we show that involution-like regression of epithelium occurs, at least in part, in an epithelium-intrinsic manner. Our observations do not contradict the crucial role of paracrine signaling required for proper involution, including the leukemia inhibitory factor and TGFβ signaling that activate STAT3-mediated regression of epithelium (Nguyen and Pollard, 2000; Kritikou et al., 2003; Hughes and Watson, 2012). Our results point to the existence of epithelial-intrinsic mechanisms of involution, for study of which our epithelial-only organoid model could be advantageous. Certainly, more work is required to establish this model as a valid system for studying physiological involution. In this study, we did not evaluate the onset of programmed cell death and its regulation. In addition, optimization of the culture conditions with cytokine cocktail would be required to further mimic physiological involution.

Several human diseases, developmental defects, or insufficiencies in mammary epithelial tissue are linked to lactation and involution period. Among others, inadequate milk production affects many women after giving birth, especially after premature deliveries and with obese mothers (Olsen and Gordon, 1990; Kent et al., 2012; Nommsen-Rivers, 2016). We propose that human breast tissue, gained from reduction mammoplasties, could be utilized to isolate primary human breast organoids for an analogous lactation assay. Furthermore, findings from murine organoids could be translated into human organoids to identify physiological barriers for lactation, which will provide valuable information for developing novel interventions to support lactation success and provide health benefit across two generations. Moreover, our organoid model could be used to investigate mechanisms of pregnancy-associated breast cancer, an aggressive form of breast cancer with peak of incidence within 5 years after delivery (Schedin, 2006). Mammary organoids isolated from genetic mouse models, such as animals carrying mutations in oncogenes or tumor suppressors, or organoids exposed to carcinogens could be used in our lactation model to unveil mechanisms and signaling pathways leading to epithelial cell carcinogenesis.

## Data Availability Statement

The raw data supporting the conclusions of this article will be made available by the authors, without undue reservation, to any qualified researcher.

## Ethics Statement

The animal study was reviewed and approved by the French legislation in compliance with European Communities Council Directives (A 75-15-01-3), the regulations of Institut Pasteur Animal Care Committees (CETEA),the Ministry of Agriculture of the Czech Republic, and the Expert Committee for Laboratory Animal Welfare at the Faculty of Medicine, Masaryk University.

## Author Contributions

JS, AC, EC, and ZK performed the experimental work. AC, JS, ZK, and HL contributed to the experimental design and data analysis. AC, ZK, and HL supervised the study. All the authors interpreted the data. ZK and HL acquired funding for the study. AC, JS, and ZK wrote the manuscript. All authors discussed the results and approved the final version of the manuscript.

## Conflict of Interest

The authors declare that the research was conducted in the absence of any commercial or financial relationships that could be construed as a potential conflict of interest.

## Abbreviations

BOM: basal organoid medium
Csn2: Casein2– β-casein gene
ECM: extracellular matrix
EGF: epidermal growth factor
FGF2,7: or 10, fibroblast growth factor 2, 7 or 10
LM: lactation medium
Mmp: matrix metalloproteinase
TGF α: transforming growth factor- α
TGF β: transforming growth factor- β
Wap: whey acidic protein.

## References

[B1] Ackland M. L. Ward J. Ackland C. M. Greaves M. Walker M. (2003). Extracellular matrix induces formation of organoids and changes in cell surface morphology in cultured human breast carcinoma cells PMC42-LA. *In Vitro Cell. Dev. Biol. Anim.* 39 428–433. 10.1290/1543-706X2003039 14658925

[B2] Adriance M. C. Inman J. L. Petersen O. W. Bissell M. J. (2005). Myoepithelial cells: good fences make good neighbors. *Breast Cancer Res.* 7 190–197. 10.1186/bcr1286 16168137 PMC1242144

[B3] Aoki M. Wartenberg P. Grünewald R. Phillipps H. R. Wyatt A. Grattan D. R. (2019). Widespread Cell-specific prolactin receptor expression in multiple murine organs. *Endocrinology* 160 2587–2599. 10.1210/en.2019-2234 31373638

[B4] Artegiani B. Clevers H. (2018). Use and application of 3D-organoid technology. *Hum. Mol. Genet.* 27 R99–R107. 10.1093/hmg/ddy187 29796608

[B5] Brisken C. O’Malley B. (2010). Hormone action in the mammary gland. *Cold Spring Harb. Perspect. Biol.* 2:a003178. 10.1101/cshperspect.a003178 20739412 PMC2982168

[B6] Brisken C. Rajaram R. D. (2006). Alveolar and lactogenic differentiation. *J. Mammary Gland Biol. Neoplasia* 11 239–248. 10.1007/s10911-006-9026-9020 17111223

[B7] Campbell J. J. Botos L.-A. Sargeant T. J. Davidenko N. Cameron R. E. Watson C. J. (2014). A 3-D in vitro co-culture model of mammary gland involution. *Integr. Biol. Quant. Biosci. Nano Macro.* 6 618–626. 10.1039/c3ib40257f 24722402

[B8] Ewald A. J. Brenot A. Duong M. Chan B. S. Werb Z. (2008). Collective epithelial migration and cell rearrangements drive mammary branching morphogenesis. *Dev. Cell* 14 570–581. 10.1016/j.devcel.2008.03.003 18410732 PMC2773823

[B9] Freestone D. Cater M. A. Ackland M. L. Paterson D. Howard D. L. de Jonge M. D. (2014). Copper and lactational hormones influence the CTR1 copper transporter in PMC42-LA mammary epithelial cell culture models. *J. Nutr. Biochem.* 25 377–387. 10.1016/j.jnutbio.2013.11.011 24485600

[B10] Froemke R. C. Carcea I. (2017). “Chapter 13 - oxytocin and brain plasticity,” in *Principles of Gender-Specific Medicine*, ed. Legato M. J. (San Diego: Academic Press), 161–182. 10.1016/B978-0-12-803506-1.00037-1

[B11] Goodwin K. Nelson C. M. (2018). Myoepithelial crowd control of cancer cells. *J. Cell Biol.* 217 3319–3321. 10.1083/jcb.201808097 30194268 PMC6168265

[B12] Green K. A. Lund L. R. (2005). ECM degrading proteases and tissue remodelling in the mammary gland. *Bioessays News Rev. Mol. Cell. Dev. Biol.* 27 894–903. 10.1002/bies.20281 16108064

[B13] Haaksma C. J. Schwartz R. J. Tomasek J. J. (2011). Myoepithelial cell contraction and milk ejection are impaired in mammary glands of mice lacking smooth muscle alpha-actin. *Biol. Reprod.* 85 13–21. 10.1095/biolreprod.110.090639 21368298 PMC3123380

[B14] Hennighausen L. Robinson G. W. (2005). Information networks in the mammary gland. *Nat. Rev. Mol. Cell Biol.* 6 715–725. 10.1038/nrm1714 16231422

[B15] Huch M. Koo B.-K. (2015). Modeling mouse and human development using organoid cultures. *Development* 142 3113–3125. 10.1242/dev.118570 26395140

[B16] Huebner R. J. Neumann N. M. Ewald A. J. (2016). Mammary epithelial tubes elongate through MAPK-dependent coordination of cell migration. *Development* 143 983–993. 10.1242/dev.127944 26839364 PMC4813284

[B17] Hughes K. Watson C. J. (2012). The spectrum of STAT functions in mammary gland development. *JAKSTAT* 1 151–158. 10.4161/jkst.19691 24058764 PMC3670238

[B18] Jamieson P. R. Dekkers J. F. Rios A. C. Fu N. Y. Lindeman G. J. Visvader J. E. (2017). Derivation of a robust mouse mammary organoid system for studying tissue dynamics. *Development* 144 1065–1071. 10.1242/dev.145045 27993977

[B19] Jena M. K. Jaswal S. Kumar S. Mohanty A. K. (2019). Molecular mechanism of mammary gland involution: an update. *Dev. Biol.* 445 145–155. 10.1016/j.ydbio.2018.11.002 30448440

[B20] Kent J. C. Prime D. K. Garbin C. P. (2012). Principles for Maintaining or Increasing Breast Milk Production. *J. Obstet. Gynecol. Neonatal Nurs.* 41 114–121. 10.1111/j.1552-6909.2011.01313.x 22150998

[B21] Kim S.-H. Wu S.-Y. Baek J.-I. Choi S. Y. Su Y. Flynn C. R. (2015). A post-developmental genetic screen for zebrafish models of inherited liver disease. *PLoS One* 10:e0125980. 10.1371/journal.pone.0125980 25950913 PMC4423964

[B22] Knight C. H. Maltz E. Docherty A. H. (1986). Milk yield and composition in mice: effects of litter size and lactation number. *Comp. Biochem. Physiol. A* 84 127–133. 10.1016/0300-9629(86)90054-x 2871967

[B23] Koledova Z. (2017a). 3D cell culture: an introduction. *Methods Mol. Biol.* 1612 1–11. 10.1007/978-1-4939-7021-6-1 28634931

[B24] Koledova Z. (2017b). 3D coculture of mammary organoids with fibrospheres: a model for studying epithelial-stromal interactions during mammary branching morphogenesis. *Methods Mol. Biol.* 1612 107–124. 10.1007/978-1-4939-7021-6-8 28634938

[B25] König B. Markl H. (1987). Maternal care in house mice. *Behav. Ecol. Sociobiol.* 20 1–9. 10.1007/BF00292161

[B26] Koopman R. Schaart G. Hesselink M. K. (2001). Optimisation of oil red O staining permits combination with immunofluorescence and automated quantification of lipids. *Histochem. Cell Biol.* 116 63–68. 10.1007/s004180100297 11479724

[B27] Kritikou E. A. Sharkey A. Abell K. Came P. J. Anderson E. Clarkson R. W. E. (2003). A dual, non-redundant, role for LIF as a regulator of development and STAT3-mediated cell death in mammary gland. *Development* 130 3459–3468. 10.1242/dev.00578 12810593

[B28] Linnemann J. R. Miura H. Meixner L. K. Irmler M. Kloos U. J. Hirschi B. (2015). Quantification of regenerative potential in primary human mammary epithelial cells. *Development* 142 3239–3251. 10.1242/dev.123554 26071498 PMC4582177

[B29] Lu P. Ewald A. J. Martin G. R. Werb Z. (2008). Genetic mosaic analysis reveals FGF receptor 2 function in terminal end buds during mammary gland branching morphogenesis. *Dev. Biol.* 321 77–87. 10.1016/j.ydbio.2008.06.005 18585375 PMC2582391

[B30] Lund L. R. Rømer J. Thomasset N. Solberg H. Pyke C. Bissell M. J. (1996). Two distinct phases of apoptosis in mammary gland involution: proteinase-independent and -dependent pathways. *Dev. Camb. Engl.* 122 181–193. 8565829 10.1242/dev.122.1.181 PMC2933211

[B31] Macias H. Hinck L. (2012). Mammary gland development. *Wiley Interdiscip. Rev. Dev. Biol.* 1 533–557. 10.1002/wdev.35 22844349 PMC3404495

[B32] Mroue R. Inman J. Mott J. Budunova I. Bissell M. J. (2015). Asymmetric expression of connexins between luminal epithelial- and myoepithelial- cells is essential for contractile function of the mammary gland. *Dev. Biol.* 399 15–26. 10.1016/j.ydbio.2014.11.026 25500615 PMC4996272

[B33] Neumann N. M. Perrone M. C. Veldhuis J. H. Huebner R. J. Zhan H. Devreotes P. N. (2018). Coordination of receptor tyrosine kinase signaling and interfacial tension dynamics drives radial intercalation and tube elongation. *Dev. Cell* 45 67–82.e6. 10.1016/j.devcel.2018.03.011 29634937 PMC5983037

[B34] Nguyen A. V. Pollard J. W. (2000). Transforming growth factor beta3 induces cell death during the first stage of mammary gland involution. *Development* 127 3107–3118. 10862748 10.1242/dev.127.14.3107

[B35] Nishimori K. Young L. J. Guo Q. Wang Z. Insel T. R. Matzuk M. M. (1996). Oxytocin is required for nursing but is not essential for parturition or reproductive behavior. *Proc. Natl. Acad. Sci. U.S.A.* 93 11699–11704. 10.1073/pnas.93.21.11699 8876199 PMC38121

[B36] Nommsen-Rivers L. A. (2016). Does insulin explain the relation between maternal obesity and poor lactation outcomes? an overview of the literature. *Adv. Nutr.* 7 407–414. 10.3945/an.115.011007 26980825 PMC4785481

[B37] Olsen C. G. Gordon R. E. (1990). Breast disorders in nursing mothers. *Am. Fam. Phys.* 41 1509–1516. 2333828

[B38] Parsa S. Ramasamy S. K. De Langhe S. Gupte V. V. Haigh J. J. Medina D. (2008). Terminal end bud maintenance in mammary gland is dependent upon FGFR2b signaling. *Dev. Biol.* 317 121–131. 10.1016/j.ydbio.2008.02.014 18381212

[B39] Qu Y. Han B. Gao B. Bose S. Gong Y. Wawrowsky K. (2017). Differentiation of human induced pluripotent stem cells to mammary-like organoids. *Stem Cell Rep.* 8 205–215. 10.1016/j.stemcr.2016.12.023 28132888 PMC5312254

[B40] Raymond K. Cagnet S. Kreft M. Janssen H. Sonnenberg A. Glukhova M. A. (2011). Control of mammary myoepithelial cell contractile function by α3β1 integrin signalling. *EMBO J.* 30 1896–1906. 10.1038/emboj.2011.113 21487391 PMC3098485

[B41] Schedin P. (2006). Pregnancy-associated breast cancer and metastasis. *Nat. Rev. Cancer* 6 281–291. 10.1038/nrc1839 16557280

[B42] Shamir E. R. Ewald A. J. (2014). Three-dimensional organotypic culture: experimental models of mammalian biology and disease. *Nat. Rev. Mol. Cell Biol.* 15 647–664. 10.1038/nrm3873 25237826 PMC4352326

[B43] Simian M. Hirai Y. Navre M. Werb Z. Lochter A. Bissell M. J. (2001). The interplay of matrix metalloproteinases, morphogens and growth factors is necessary for branching of mammary epithelial cells. *Development* 128 3117–3131. 11688561 10.1242/dev.128.16.3117 PMC2785713

[B44] Sirka O. K. Shamir E. R. Ewald A. J. (2018). Myoepithelial cells are a dynamic barrier to epithelial dissemination. *J. Cell Biol.* 217 3368–3381. 10.1083/jcb.201802144 30061105 PMC6168248

[B45] Stein T. Salomonis N. Gusterson B. A. (2007). Mammary gland involution as a multi-step process. *J. Mammary Gland Biol. Neoplasia* 12 25–35. 10.1007/s10911-007-9035-9037 17431797

[B46] Sternlicht M. D. (2006). Key stages in mammary gland development: the cues that regulate ductal branching morphogenesis. *Breast Cancer Res.* 8:201. 10.1186/bcr1368 16524451 PMC1413974

[B47] Stewart T. A. Hughes K. Stevenson A. J. Marino N. Ju A. L. Morehead M. (2019). Mammary mechanobiology: PIEZO1 mechanically-activated ion channels in lactation and involution. *bioRxiv* [Preprint], 10.1101/649038 33262312

[B48] Sumbal J. Koledova Z. (2019). FGF signaling in mammary gland fibroblasts regulates multiple fibroblast functions and mammary epithelial morphogenesis. *Development* 146:dev185306. 10.1242/dev.185306 31699800

[B49] Vorherr H. Vorherr U. F. Solomon S. (1978). Contamination of prolactin preparations by antidiuretic hormone and oxytocin. *Am. J. Physiol.* 234 F318–F324. 10.1152/ajprenal.1978.234.4.F318 565596

[B50] Watson C. J. (2006). Key stages in mammary gland development - involution: apoptosis and tissue remodelling that convert the mammary gland from milk factory to a quiescent organ. *Breast Cancer Res.* 8:203. 10.1186/bcr1401 16677411 PMC1557708

[B51] Xian W. Schwertfeger K. L. Vargo-Gogola T. Rosen J. M. (2005). Pleiotropic effects of FGFR1 on cell proliferation, survival, and migration in a 3D mammary epithelial cell model. *J. Cell Biol.* 171 663–673. 10.1083/jcb.200505098 16301332 PMC2171554

[B52] Zwick R. K. Rudolph M. C. Shook B. A. Holtrup B. Roth E. Lei V. (2018). Adipocyte hypertrophy and lipid dynamics underlie mammary gland remodeling after lactation. *Nat. Commun.* 9:3592. 10.1038/s41467-018-05911-5910 30181538 PMC6123393

