# Supplementary material for: Primary Mammary Organoid Model of Lactation and Involution
Source: Front Cell Dev Biol. 2020 Mar 19;8:68. doi: 10.3389/fcell.2020.00068 (PMC7098375; doi:10.3389/fcell.2020.00068)
Supplement: Supplementary file 1 [file Data_Sheet_1.PDF]

Supplementary Material

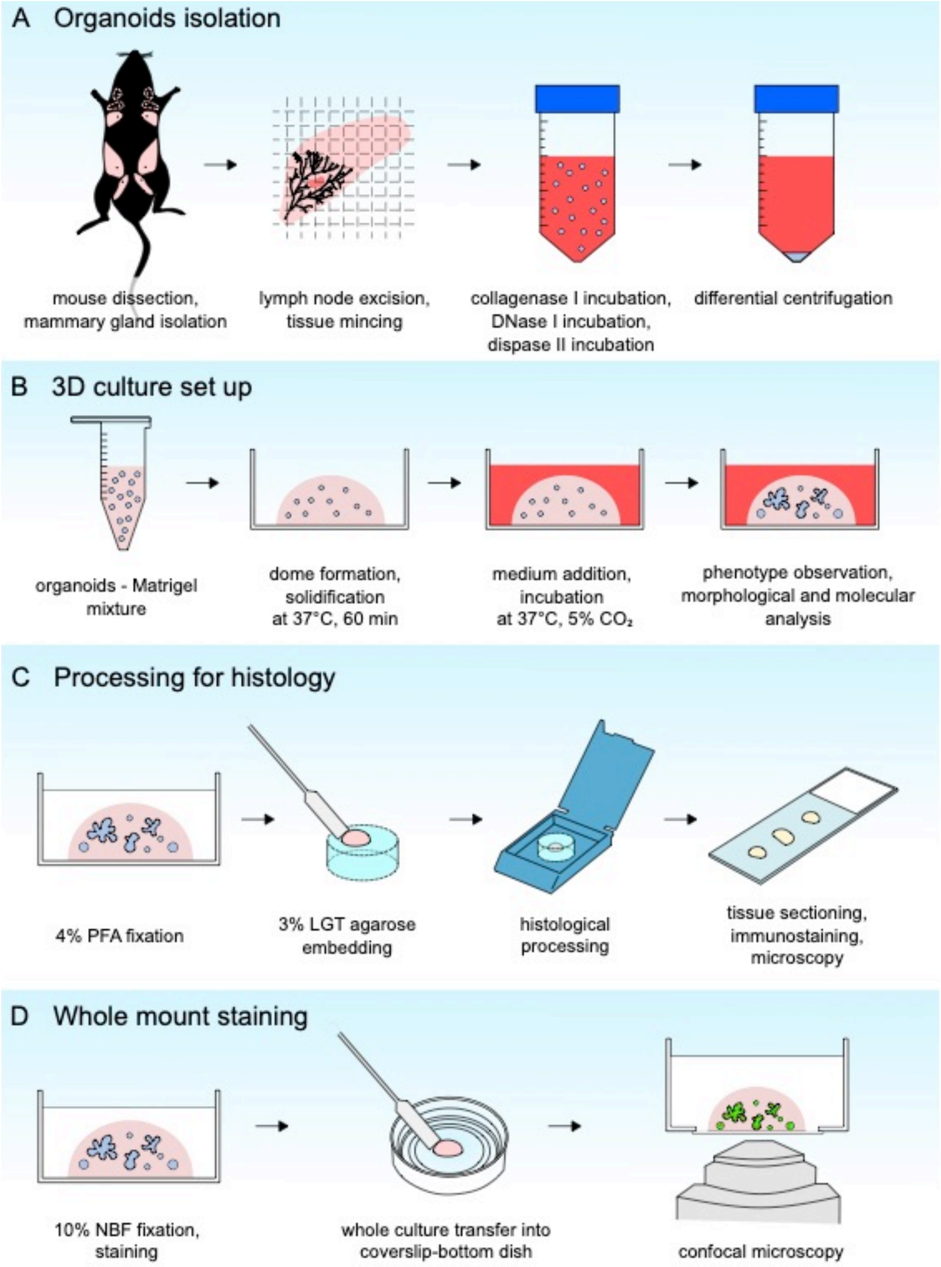

SUPPLEMENTARY FIGURE 1 | Schematic depicting organoid isolation and processing. **(A)** Mammary glands number 3, 4 and 5 are dissected from mouse cadaver, minced with scalpels, digested with enzymes and epithelial organoids are isolated from stromal fraction by differential centrifugation. **(B)** Organoids are mixed with Matrigel on ice, a dome-shape structure is formed in a well of 24-well plate. Solidified Matrigel is then overlaid with culture medium and cultured at 37°C and 5% CO_2. **(C)** For histological analysis, Matrigel with grown organoids is fixed and washed in the well, transferred to agarose, processed for histology, embedded in paraffin, cut on microtome and used in desired staining protocol. **(D)** For whole mount staining of the 3D culture, Matrigel with grown organoids is fixed, washed and stained in the well. Then the whole culture is transferred to a coverslip-bottom dish with a drop of agarose, and organoids are imaged with a confocal microscope.

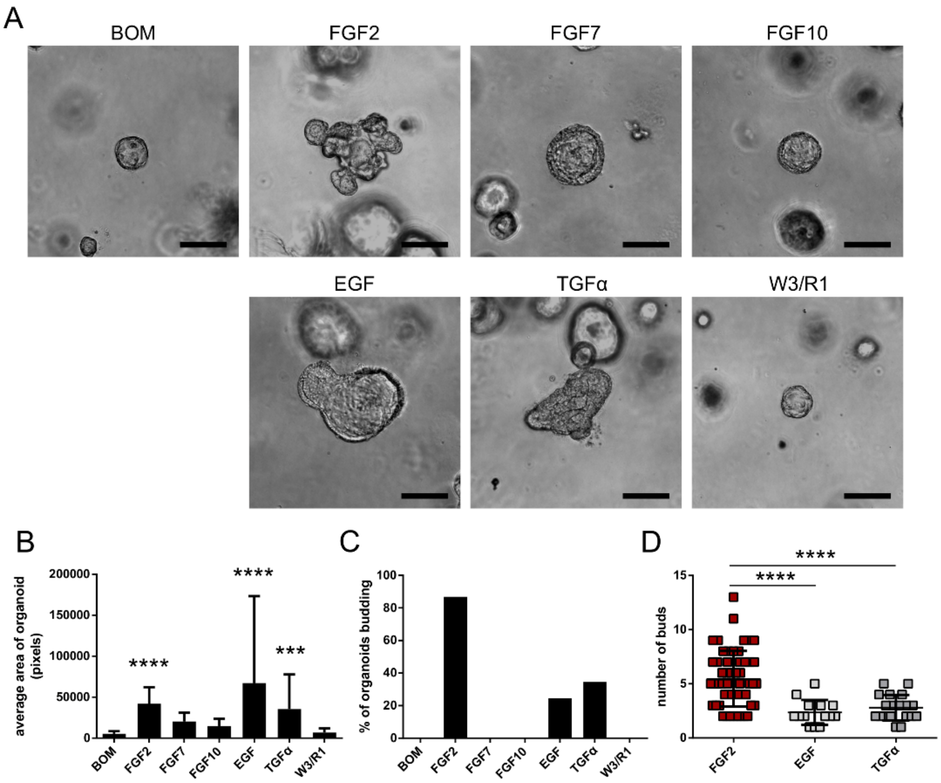

SUPPLEMENTARY FIGURE 2 | FGF2 is a potent inducer of organoid branching morphogenesis**. (A)** Bright-field images of organoids after 7 days of culture in BOM or in BOM supplemented with 2.5 nM FGF2, 2.5 nM FGF7, 2.5 nM FGF10, 50 ng/ml EGF, 5 nM TGFα, or a combination of 10 ng/ml WNT3A and 50 ng/ml R-spondin 1 (W3/R1). Scale bars represent 100 µm. **(B)** Quantification of organoid size after 7 days of culture with different growth factors. The plot shows mean + SD; n = 1, N = 55-78 organoids per condition. One-way ANOVA, *** $p < 0.001$, **** $p < 0.0001$. (C, D) Quantification of number of budding organoids **(C)** and the number of buds per budding organoid **(D)**. The plots show mean or mean ± SD, n = 1, N = 14 – 52 budding organoids

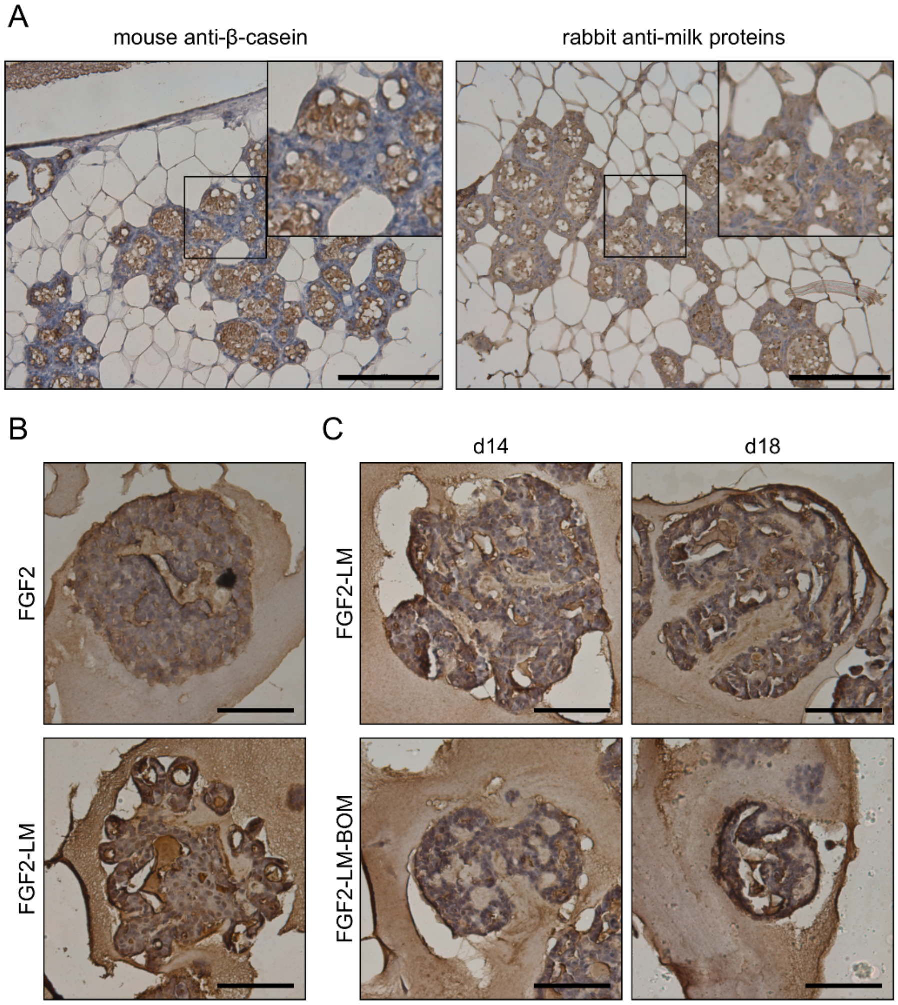

SUPPLEMENTARY FIGURE 3 | Detection of milk proteins using immunohistochemistry **(A)** Mammary gland tissue on day 1 of lactation stained with mouse antibody against β-casein or rabbit antibody against milk proteins. **(B)** Organoids after 6 days of FGF2 treatment before (FGF2) or after 4 day-long LM treatment (FGF2-LM). **(C)** Organoids on day 14 and 18 of culture with continuous LM treatment (FGF2-LM) or LM withdrawal and replacement with BOM (FGF2-LM-BOM). Scale bars represent 100 μm.

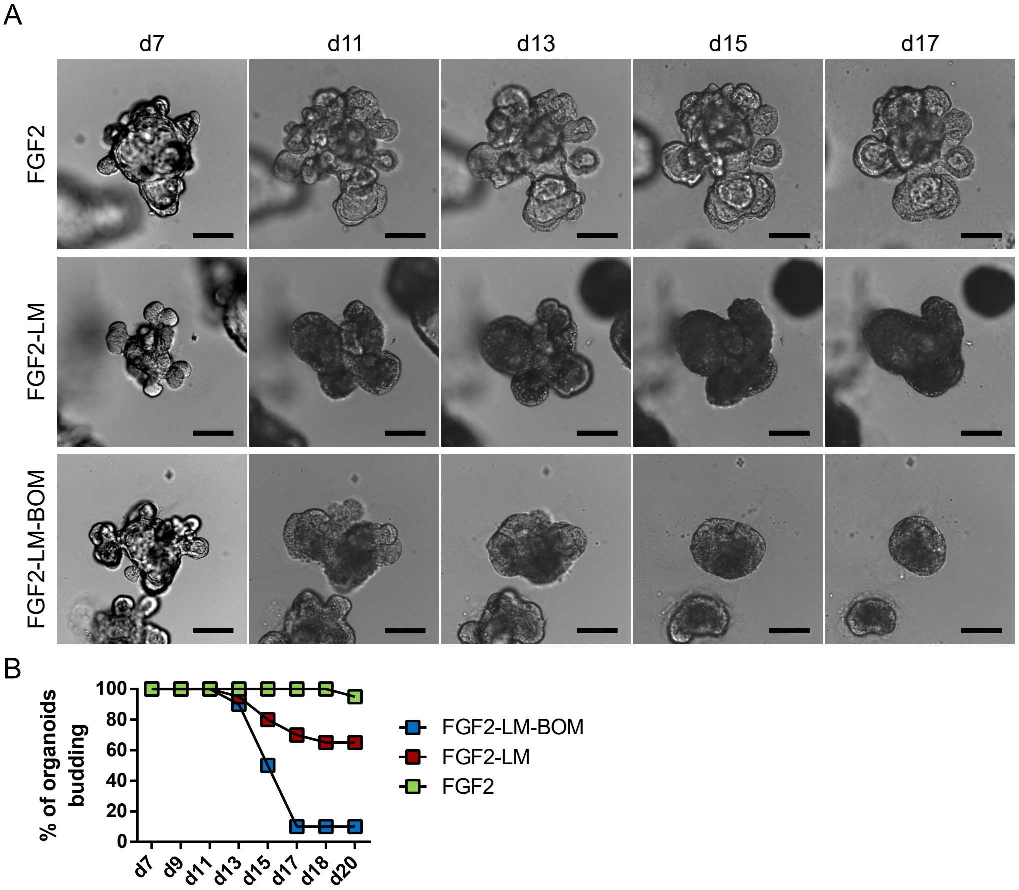

SUPPLEMENTARY FIGURE 4 | Long-term culture with FGF2 conserves branched epithelial morphology. (A) Bright-field images from time-lapse imaging of organoid morphogenesis under continuous FGF2 treatment (FGF2), continuous LM treatment (LM) or LM withdrawal and replacement with BOM (LM-BOM). Scale bars represent 100 µm. **(B)** Quantification of the number of organoids with "budding" morphology.

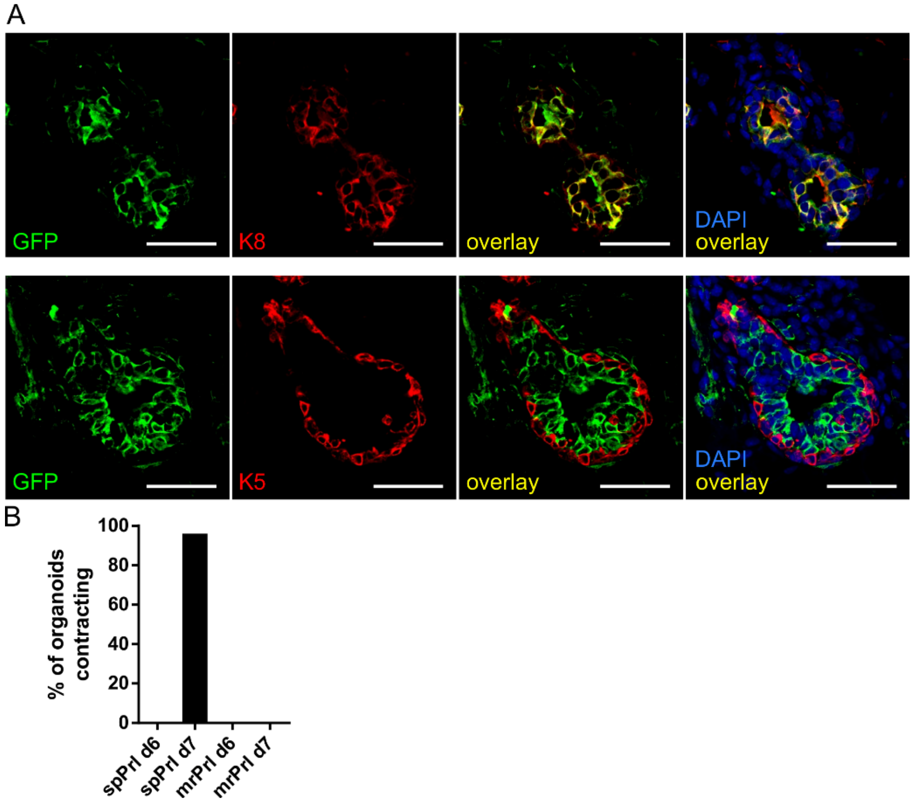

SUPPLEMENTARY FIGURE 5 | Contraction of organoids is not caused by direct prolactin signaling. **(A)** Immunofluorescent staining of mammary gland from *Prlr-IRES-Cre;ROSA26-CAGS-GFP* mouse. Green, GFP in cells expressing prolactin receptor; red, keratin 5 (K5) or keratin 8 (K8); blue, DAPI). Scale bars represent 50 µm. **(B)** Quantification of contracting organoids from movies recorded on day 6 (before LM treatment) and day 7 (after LM treatment). Sheep pituitary (spPRL) or mouse recombinant prolactin (mrPRL) were used to prepare LM.
