# Supplementary material for: Primary Mammary Organoid Model of Lactation and Involution
Source: Front Cell Dev Biol. 2020 Mar 19;8:68. doi: 10.3389/fcell.2020.00068 (PMC7098375; doi:10.3389/fcell.2020.00068)
Supplement: Supplementary file 2 [file Presentation_1.pptx]

## Slide 1
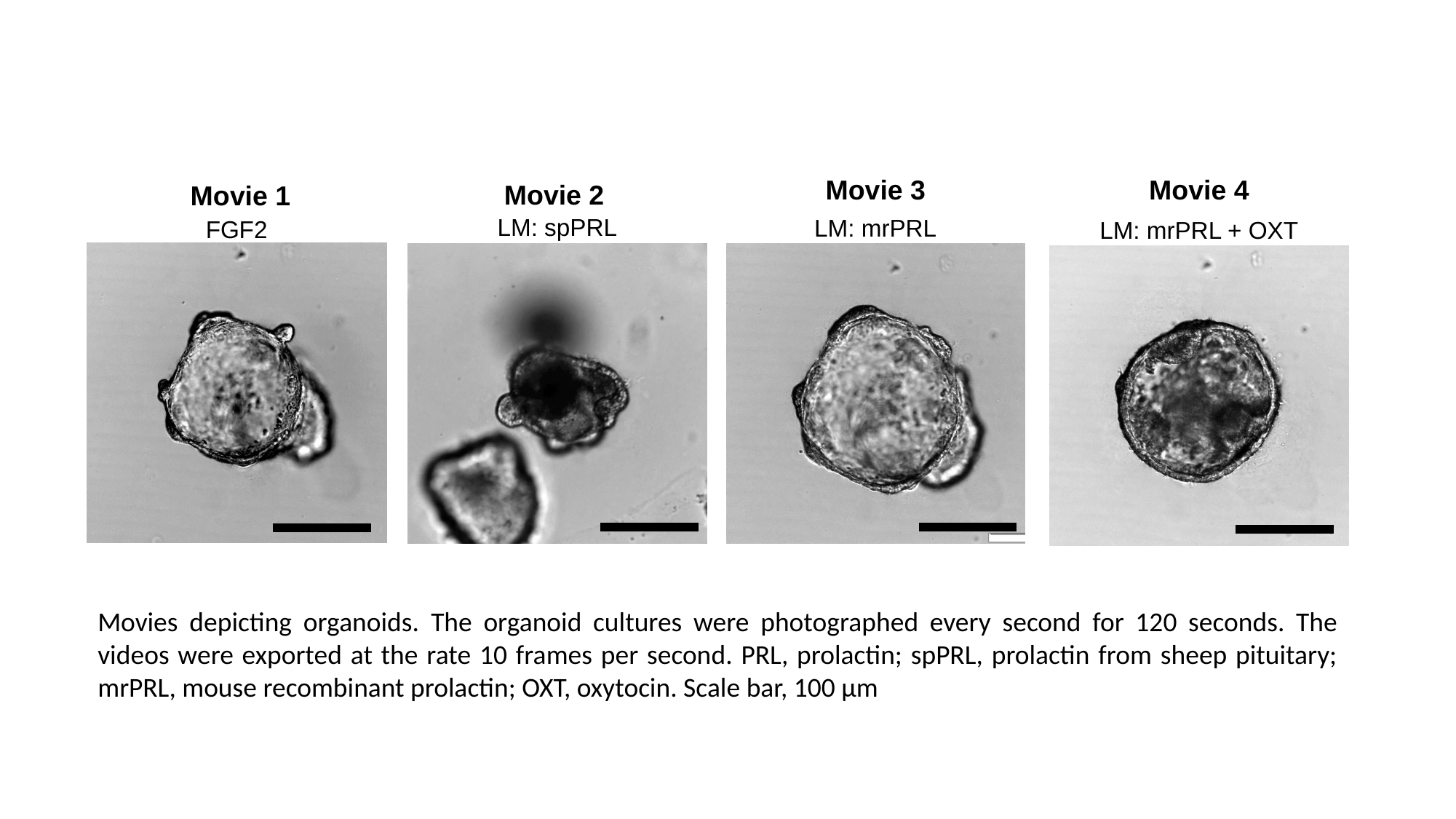

Movie 3
Movie 4
Movie 2
Movie 1
LM: spPRL
LM: mrPRL
FGF2
LM: mrPRL + OXT
Movies depicting organoids. The organoid cultures were photographed every second for 120 seconds. The videos were exported at the rate 10 frames per second. PRL, prolactin; spPRL, prolactin from sheep pituitary; mrPRL, mouse recombinant prolactin; OXT, oxytocin. Scale bar, 100 μm
